# Incidence, risk factors and outcomes of checkpoint inhibitor-induced liver injury: A 10-year real-world retrospective cohort study

**DOI:** 10.1016/j.jhepr.2023.100851

**Published:** 2023-07-18

**Authors:** Edmond Atallah, Sarah J. Welsh, Brent O’Carrigan, Ana Oshaughnessy, Igboin Dolapo, Andrew S. Kerr, Joanna Kucharczak, Colin Y.C. Lee, Colin Crooks, Amy Hicks, Chenchu Ramu Chimakurthi, Ankit Rao, Hester Franks, Poulam M. Patel, Guruprasad P. Aithal

**Affiliations:** 1 Nottingham Digestive Diseases Centre, Translational Medical Sciences, School of Medicine, University of Nottingham, Nottingham, UK; 2 National Institute for Health Research (NIHR) Nottingham Biomedical Research Centre, Nottingham University Hospitals NHS Trust and the University of Nottingham, Nottingham, UK; 3 Department of Oncology, Cambridge University Hospitals NHS Foundation Trust, Cambridge, UK; 4 Department of Oncology, Nottingham University Hospitals NHS Trust, Nottingham, UK; 5 Leeds Liver Unit, Leeds Teaching Hospitals NHS Trust, Leeds, UK; 6 Centre for Cancer Sciences, Translational Medical Sciences, Biodiscovery Institute, University of Nottingham, Nottingham, UK

**Keywords:** Checkpoint inhibitors, Immunotherapy, Immune-mediated hepatitis, Hepatotoxicity, Drug-induced liver injury, Incidence rate, Risk factors

## Abstract

**Background & Aims:**

Checkpoint inhibitors (CPI) account for increasing numbers of drug-induced liver injury (DILI) cases. We aimed to determine the incidence rate and risk factors associated with checkpoint inhibitor-induced liver injury (ChILI).

**Methods:**

Prescription event monitoring was performed on all melanoma and renal cancer patients who received CPI at a tertiary centre between 2011 and 2021. ChILI cases were identified using the definitions, grading, and causality assessment methods validated for DILI. We assessed risk factors associated with ChILI in CPI-naive patients using multivariable logistic regression model. Consecutive patients with suspected ChILI from two other tertiary centres were adjudicated and combined for case characterisation and outcomes of ChILI.

**Results:**

Out of 432 patients who received CPI over 10 years, ChILI occurred in 38 (8.8%) with an overall incidence rate of 11.5 per 1,000 person-months (95% CI 8.2–15.8). Probability of ChILI was highest in combination therapy (32%) and no new events occurred beyond 135 days of treatment. Risk factor analysis showed that combination therapy, female sex, higher baseline alanine transferase level and lower baseline alkaline phosphatase level were independently associated with higher risk of ChILI. In total, 99 patients were adjudicated to have ChILI from three centres. Although Common Terminology Criteria for Adverse Events classified 20 patients (20.2%) to have ‘life-threatening’ grade 4 hepatitis, ChILI severity was graded as mild in 45 (45.5%) and moderate in the remaining 54 (54.5%) cases.

**Conclusions:**

The real-world risk of ChILI is higher than previously reported. Among patients receiving dual CPI, this risk falls markedly after 4.5 months. As Common Terminology Criteria for Adverse Events overestimates its clinical severity, case-definition, evaluation and management of ChILI should be revised to harmonise care.

**Impact and implications:**

Using prescription event monitoring over a 10-year period, the incidence rate of checkpoint inhibitor induced liver injury (ChILI) based on established case definitions for drug-induced liver injury (DILI) is 11.5 per 1,000 person-months. Formal causality assessment identified an alternative cause in 19% of patients with suspected ChILI highlighting the importance of systematic evaluation by clinicians to minimise unnecessary immunosuppression. Intensity of monitoring in patients receiving combination therapy regime after 4.5 months of therapy can be reduced as the risk of new onset ChILI beyond this point is minimal. Current Common Terminology Criteria for Adverse Events (CTCAE) grading overestimates clinical severity of ChILI and hence contributes to avoidable hospitalisation.

## Highlights

• Real-world risk of checkpoint inhibitor-induced liver injury (ChILI) is higher than previously reported.
• In patients receiving a combination therapy regimen, the risk of new onset ChILI beyond 4.5 months of therapy is minimal.
• Female sex, lower baseline ALP and higher ALT are independent factors associated with increased risk of ChILI.
• Severity of ChILI using DILI criteria is lower than that estimated by the CTCAE criteria used in oncology.
• Rechallenge following ChILI is feasible; less than 10% develop recurrent liver injury that meet DILI criteria.

## Graphical abstract

**Figure:**
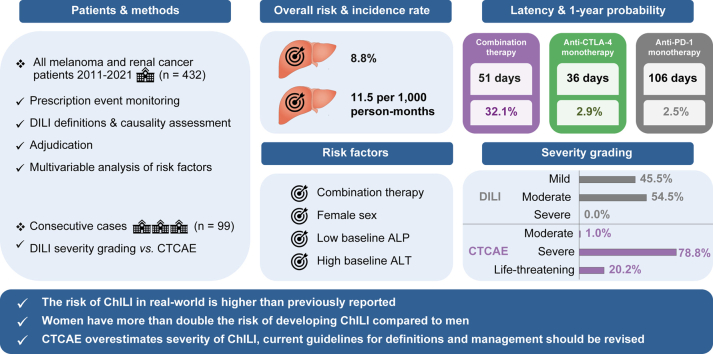

## Introduction

The use of checkpoint inhibitors (CPI) has increased exponentially following their remarkable efficacy improving survival in many advanced malignancies. In patients with advanced melanoma or renal cell carcinoma (RCC), treatment with CPI targeting programmed cell death protein 1 (PD1) (e.g. nivolumab, pembrolizumab) as monotherapy or in combination with anti-cytotoxic T-lymphocyte-associated protein 4 (CTLA4) (ipilimumab with nivolumab) are now established as standard of care.[1], [2], [3] With their adoption as an effective therapy for advanced cancers, a range of immune-related adverse events (irAE) affecting different organs have also emerged.

Checkpoint inhibitor-induced liver injury (ChILI) is one of the important irAE following checkpoint blockade.^4^ Among fatalities reported in patients receiving anti-PD1 or anti-programmed death ligand 1 therapy, 22% (77/333) were attributed to ‘hepatitis’.^5^ CPI account for increasing proportions of drug-induced liver injury (DILI) cases in recent cohort studies.^6^^,^^7^ Although published studies have used the definition and grading based on the Common Terminology Criteria for Adverse Events (CTCAE), a standardised toxicity grading system widely accepted in clinical trials of cancer therapy,^8^^,^^9^ this system was developed prior to the era of immunotherapy and there are limitations to its applicability for immunotherapy and irAE.^10^ The CTCAE classifies all organ system toxicities by severity grades 1 to 5 (grade 1 is mild, grade 2 is moderate, grade 3 is severe or medically significant, grade 4 refers to life-threatening, and grade 5 refers to fatal toxicity). For hepatic toxicity, these grades do not reflect clinical severity; for example, 20-fold elevation of either alanine aminotransferase (ALT), aspartate aminotransferase (AST), or alkaline phosphatase (ALP) alone is classified as life-threatening (grade 4) hepatitis without evidence of any hepatic functional impairment such jaundice, coagulopathy, ascites, or encephalopathy. In addition, these events have not been assessed using validated causality assessment methods used for DILI. A recent study evaluated 70 events of liver injury identified based on liver biochemistry among 491 patients on pembrolizumab and adjudicated only 20 (28.6%) of these as ChILI.^11^ Therefore, identification and formal evaluation of suspected ChILI using the definition, severity grading, and causality assessment using validated tools should form a basis for clinical practice guidance for the management of patients receiving CPI.^12^

The incidence of ChILI has been reported using incidence proportion (risk) which varies widely in the literature. The overall risk of CTCAE grade 3 and 4 liver toxicity reported in clinical trials ranges from 0.6 to 16% depending on cancer and CPI regime or dose.^4^^,^^13^ In systematic reviews of randomised trials of CPI, the reported pooled risk of ≥ grade 3 hepatotoxicity following anti-CTLA4 and anti-PD1 was 3.3% and 9%.^9^^,^^14^ However, retrospective studies reported much higher rates, 23.1% of all patients receiving CPI,^15^ and 21–33% of melanoma patients receiving combination therapy.^16^^,^^17^ Incidence proportion uses the number of patients starting CPI at the start of study as a denominator, it does not consider the difference in durations of exposure to CPI between patients which varies significantly in clinical practice. In contrast, the incidence rate takes the number of patients and their time at risk into account and is considered a more accurate epidemiological method of event frequency, especially in dynamic populations with long follow-up. To date, no studies have defined ChILI and graded its severity according to DILI definitions or accurately assessed its incidence risk per person-time according to cancer type and CPI regime.

There are also limited data on risk factors that increase or decrease the development of ChILI during CPI therapy. Although recent studies showed that some clinical characteristics and baseline blood profile may predict immune-related adverse events and clinical outcomes following CPI,[18], [19], [20] and represent possible risk factors of hepatotoxicity following CPI,^17^^,^^21^ formal association between risk factors and ChILI have not been investigated using multivariable modelling.

We aimed to calculate the risk, incidence rate, and cumulative incidence of ChILI in patients with melanoma and RCC receiving CPI over a 10-year period. We have identified risk factors associated with ChILI occurrence in CPI-naive patients. We have also characterised ChILI cases from three centres and reported their clinical outcomes of rechallenge.

## Patients and methods

### Patient populations

All patients with malignant melanoma or advanced RCC who received at least one cycle of CPI (without concurrent chemotherapy or other targeted therapy) at Nottingham University Hospitals (NUH) from 2011 to June 2021 were included and categorised into seven subgroups based on cancer and CPI regime received. The project was registered at NUH, and patient informed consent was waived (21-375 C). To characterise ChILI, compare severity grading between CTCAE and DILI definitions, as well as describe outcomes of ChILI including rechallenge, we combined the cases from NUH with consecutive ChILI cases from two other UK tertiary centres. Consecutive patients with melanoma or RCC who received the same regimes and developed possible ChILI at Cambridge University Hospitals NHS Foundation Trust (CUH; study approval: ID4376/PRN10376) between October 2014 and February 2021 and Leeds Teaching Hospitals NHS Trust (LTHT; study approval: ICI) between January 2018 and December 2021 were identified and underwent adjudication.

### Outcomes and definitions

The primary outcome was ChILI occurrence which was defined and graded following the expert working group (EWG) definitions in DILI.^12^ ChILI cases were also graded following CTCAE version 5.0 to compare the risk and severity between the two grading systems.^8^ Because some patients who developed CTCAE grade 2 liver toxicity might meet EWG criteria and it is the threshold used to withhold CPI and consider corticosteroids, comparison of risk was made between EWG criteria and CTCAE grade ≥2 liver toxicity. Table S1 highlights the thresholds used to define CTCAE grades of hepatotoxicity in comparison to EWG criteria. The upper limit of normal (ULN) was defined using laboratory cut-offs. In the case of abnormal baseline liver enzymes, the definitions were adjusted to reflect change relative to the baseline rather than the ULN. Jaundice was defined as an elevation of total bilirubin (TB) ≥ 2 × ULN at any time point during the liver injury and cases who met Hy’s law (ALT ≥3 × ULN and TB ≥2 × ULN) were identified.^22^

All patients with suspected ChILI underwent formal causality assessment using the Roussel Uclaf Causality Assessment Method (RUCAM)^23^ followed by an adjudication process. Patients who developed liver injury that was likely attributable to alternative aetiology or had insufficient investigations were excluded. For ChILI cases that passed adjudication, time to occurrence from start of CPI, rate of hospitalisation, other concurrent irAE (occurred during the time of liver injury), investigations including histological features, treatment of ChILI, reported clinically significant side effects specific to steroid treatment, time to resolution (defined as normalisation of liver enzymes or return to baseline), and time to improvement to grade ≤1 CTCAE were recorded. Details of rechallenge following ChILI including regime used and outcomes were described. Recurrence of liver injury was defined based on the same EWG criteria used to define ChILI.^12^

### Prescription event monitoring to determine the risk and incidence rate of ChILI

All melanoma and RCC patients who received CPI at NUH from 2011 until June 2021 were identified using the prospective oncology prescribing database ‘Chemocare’. Liver enzymes were collected before each cycle during CPI treatment and monitored for a minimum of 3 months after stopping CPI or until December 2021. The risk of ChILI (incidence proportion) in each CPI regime was calculated by dividing the number of patients who developed ChILI by the total number of patients who received the CPI. The 95% CI for the proportion was calculated using the Wilson Score method.^24^^,^^25^

To determine the incidence rate of ChILI, the time at risk for each patient was estimated. For ChILI cases, time to event was the duration from the start of CPI regime until they met the predefined ChILI thresholds. For patients who did not develop ChILI, time at risk was defined as the duration of exposure to the specified CPI regime with latency ranges from 21 to 42 days after the last cycle, depending on the CPI regime (21 days for ipilimumab monotherapy, 28 days for nivolumab monotherapy or in combination with ipilimumab, and 42 days for pembrolizumab). The duration at risk after the last CPI cycle was based on CPI regime frequency and the available literature on the clinical pharmacokinetics and pharmacodynamics of CPI.^26^ The total person-time at risk in each subgroup was computed as person-months. The incidence rate of ChILI was estimated by dividing the number of ChILI events in each subgroup by the total person-months at risk, and 95% CI was calculated based on Byar’s method.^27^^,^^28^ The cumulative incidence (probability) of ChILI in each class of CPI was estimated using Kaplan–Meier method and cumulative incidence function.

### Case-control study to identify risk factors associated with ChILI

Patients who were adjudicated as ChILI from NUH were defined as cases; those who received CPI without developing ChILI were included in the control group. We investigated risk factors of ChILI in CPI-naive patients (receiving CPI for the first time) only, those who had CPI previously were excluded from the analysis. Haematology and liver profiles before starting CPI and clinical characteristics including age, sex, BMI, cancer type, CPI regime, and presence of liver metastases at baseline were obtained. Peripheral blood count data included absolute count of neutrophils, lymphocytes, and eosinophils, and neutrophil-to-lymphocyte ratio (NLR).

### Statistical methods

Demographic and clinical data were described using descriptive statistics, mean ± standard deviation (SD) for continuous measurements that are normally distributed, median and IQR or full range when more appropriate for non-normally distributed continuous variables. Frequencies and percentiles were used for categorical data. Differences in baseline characteristics between patients who developed ChILI and patients without ChILI were evaluated using the Student *t* test for continuous variables, or the Pearson Chi-squared test for proportions, as appropriate. Continuous variables exhibiting a skewed distribution were transformed, using the natural logarithms, before *t* tests were conducted to satisfy the prerequisite assumptions of normality. The Mann–Whitney–Wilcoxon test was applied if variables were not normally distributed despite transformation. Kruskal-Wallis one-way analysis of variance was used to assess difference of median duration of resolution of liver injury or improvement to grade 1 CTCAE between CPI classes.

To study the association between baseline blood profile and clinical characteristics with ChILI, multivariable logistic regression analysis was performed on the NUH cohort. Variables with significant difference between cases and controls were included in the final model. Age, sex, CPI regime and presence of liver metastases at baseline were considered as priori confounders and included in the final model. Odds ratios (ORs) were calculated and *p* <0.05 was considered significant. All analyses were conducted using R programme version 4.0.3 (R Foundation for Statistical Computing, Vienna, Austria).^29^ R packages used to estimate cumulative incidence were ‘survival’, ‘etm’, and ‘cmprsk’.[30], [31], [32]

## Results

### Risk, incidence rate and cumulative incidence of ChILI

**Fig. 1:**
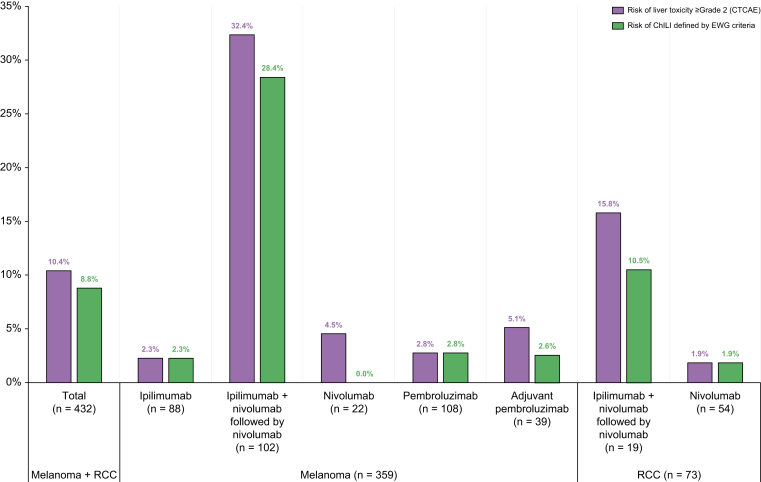
Risk of ChILI per CPI regime in melanoma and renal cancer. Risk of ChILI (incidence proportion) in each subgroup was calculated by dividing the number of patients who developed ChILI by the total number of patients who received the CPI regime. ChILI, checkpoint inhibitor-induced liver injury; CPI, checkpoint inhibitors; CTCAE, Common Terminology Criteria for Adverse Events V5.0;^8^ EWG, Expert Working Group definitions in drug-induced liver injury;^12^ RCC, renal cell carcinoma.

Over 10 years, a total of 432 patients received CPI at NUH, 359 patients with melanoma and 73 with RCC. None of the patients treated had underlying autoimmune liver disease. Fifty (11.6%) out of 432 patients had elevated ALT > ULN before starting CPI. These were investigated based on the risk factors, 15 were attributed to liver metastases and three to non-alcoholic fatty liver disease. After the first cycle of CPI, liver enzymes normalised in 23 patients (46%). The remaining nine patients had negative viral serology and autoantibody profile. Details of frequency and dose of CPI regimes are shown in Table S2. Forty-seven patients (10.9%) had acute liver injury and met the EWG criteria. However, nine out of 47 (19.1%) were excluded following causality assessment and adjudication process (seven had progressive liver metastases, one had biliary obstruction/cholangitis and one had idiosyncratic DILI). The predominant pattern of liver injury in excluded cases was cholestatic, seven out of nine cases. Overall, 38 patients developed ChILI (8.8%). On causality assessment ChILI were classified as possible in 9 (23.7%), probable in 22 (57.9%) and highly probable in 7 (18.4%). The median RUCAM score was 7 (probable). The risk of ChILI varied among CPI regimes with the highest risk occurring in melanoma patients who received combination therapy followed by anti-PD1 (28.4%) (Fig. 1). In contrast, the risk of CTCAE grade ≥2 liver toxicity was 10.4% overall and 32.4% in melanoma patients on dual CPI therapy.

**Fig. 2:**
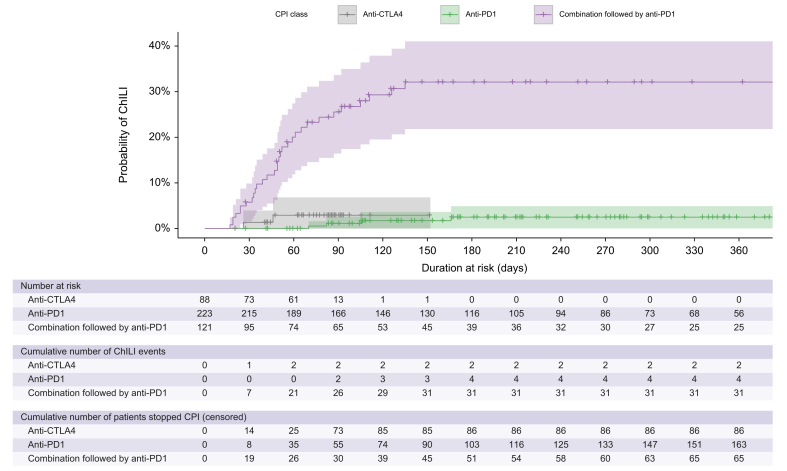
Probability of ChILI per CPI class over 1 year. Cumulative incidence (probability) of ChILI per CPI class was estimated using Kaplan–Meier method and cumulative incidence function using the R programme. Anti-CTLA4, anti-cytotoxic T-lymphocyte-associated protein 4; anti-PD1, anti-programmed cell death protein 1; ChILI, checkpoint inhibitor-induced liver injury; CPI, checkpoint inhibitors.

The overall incidence rate of ChILI was 11.5 per 1,000 person-months. The highest incidence rate was in melanoma patients receiving a combination of ipilimumab and nivolumab (38.1 per 1,000 person-months). Incidence rates of ChILI per CPI regime are shown in Table 1. The shortest latency to ChILI occurrence was observed in melanoma and renal patients who received combination therapy, median latency was 51 days (range 17–135 days). In contrast, the median latency to ChILI in patients who received anti-PD1 monotherapy was 106 days (range 70–526 days). We limited the cumulative incidence estimation of ChILI to 1 year because the number of patients at risk beyond 1 year of CPI therapy was small. In patients with melanoma and renal combined, the highest cumulative incidence (probability) of ChILI was in combination therapy, 32.1%, and no ChILI events occurred after 4.5 months from starting CPI (Fig. 2 and Table S3). In patients who received monotherapy of anti-CTLA4 or anti-PD1, the probability of developing ChILI over 1 year was 2.9% and 2.5%, respectively, as shown in Fig. 2 and Tables S4 and S5. There was one late ChILI occurrence in a patient who received anti-PD1 monotherapy after 526 days.

**Table 1:** Incidence rate of ChILI per CPI regime at NUH.

Cancer	CPI regimen	Total person-time at risk (months)	ChILI cases (n)	IR per 1,000 person-months	95% CI
Malignant melanoma	Ipilimumab	200.5	2	10.0	1.2–36
	Ipilimumab + nivolumab followed by nivolumab	761.1	29	38.1	25.5–54.7
	Nivolumab	281.2	0	0	0–13.1
	Pembrolizumab	1,035.4	3	2.9	0.6–8.5
	Adjuvant pembrolizumab	343.3	1	2.9	0.1–16.2
Advanced RCC	Ipilimumab + nivolumab followed by nivolumab	149.1	2	13.4	1.6–48.5
	Nivolumab	524.7	1	1.9	0–10.6
Total	All regimens	3,295.3	38	11.5	8.2–15.8

The incidence rate of ChILI was estimated by dividing the number of ChILI events in each subgroup by the total person-months at risk, and 95% CI was calculated based on Byar's method. CI, confidence interval; CPI, checkpoint inhibitors; ChILI, checkpoint inhibitor-induced liver injury; IR, incidence rate; RCC, renal cell carcinoma.

### Risk factors associated with ChILI in CPI-naive patients

Analysis of risk factors for patients who were treated at NUH over 10 years with no previous exposure to CPI included 36 ChILI cases and 358 controls. Clinical characteristics, baseline blood, and liver profiles of cases and controls are summarised in Table 2. Patients who developed ChILI were younger (*p* = 0.002), more likely to have received combination therapy (*p* <0.001), had significantly higher baseline ALT (*p* = 0.003), lower ALP (*p* = 0.01), lower neutrophils (*p* = 0.03) and lower NLR (*p* = 0.008). There was no significant difference in the type of cancer, BMI, presence of liver metastases, baseline lymphocytes, or eosinophils between the groups. Patients with liver metastases had multiple liver lesions throughout the liver rather than solitary lesion. In multivariate logistic regression analysis, independent risk factors associated with ChILI were combination CPI followed by anti-PD1 regime (OR 10.95; 95% CI 4.04–35.6, *p* <0.001), female sex (OR 2.54; 95% CI 1.09–6.06, *p* = 0.032), baseline ALT (1.03; 95% CI 1.01–1.05, *p* = 0.006) and ALP (0.99; 95% CI 0.98–1.00, *p* = 0.015) (Table 3).

**Table 2:** Clinical characteristics and baseline blood profile of CPI-naive patients at NUH.

	All patients (N = 394)	Cases (n = 36)	Controls (n = 358)	*p* value
Age, years; Mean, (SD)	65 (13)	57 (15)	65 (13)	**0.002**
Sex, n; (Male, %)	255 (64.7)	20 (55.6)	235 (65.6)	0.23
BMI, kg/m^2^; Median (IQR)	26 (23, 31.7)	25.5 (21.7, 31.2)	27 (23, 31.7)	0.48
Type of cancer, n (column %)				
Malignant melanoma	321 (81.5)	33 (91.7)	288 (80.4)	0.10
Advanced RCC	73 (18.5)	3 (8.3)	70 (19.6)	
Type of CPI, n (column %)				
Combination followed by anti-PD1	115 (29.2)	29 (80.5)	86 (24)	**<0.001**
Anti-CTLA4	83 (21.1)	2 (5.6)	81 (22.6)	
Anti-PD1	196 (49.7)	5 (13.9)	191 (53.4)	
Presence of liver metastases prior CPI, n (%)	61 (15.5)	5 (13.9)	56 (15.6)	0.78
ALT, IU (ULN: 45 males, 35 females); Median (IQR)	21 (14, 31)	27.5 (18, 39)	21 (14, 29)	**0.003**
ALP, IU (ULN: 130); Median (IQR)	92 (74, 124)	85 (71, 103.8)	94 (74.2, 126)	**0.01**
Total bilirubin, μmol/L (ULN: 21); Median (IQR)	9 (7, 12)	9 (7, 11.5)	9 (7, 12)	0.61
Neutrophils, × 10^9^/L: Median (IQR)	5 (3.9, 6.5)	4.4 (3.4, 5.8)	5 (4, 6.6)	**0.03**
Lymphocytes × 10^9^/L; Median (IQR)	1.6 (1.2, 2)	1.8 (1.2, 2.1)	1.5 (1.2, 2)	0.12
Neutrophil to lymphocyte ratio (NLR); Median (IQR)	3.1 (2.3, 4.8)	2.5 (2, 3.3)	3.2 (2.3, 4.9)	**0.008**
Eosinophils × 10^9^/L; Median (IQR)	0.1 (0.1, 0.2)	0.1 (0.1, 0.3)	0.1 (0.1, 0.2)	0.62∗

The baseline blood profile was unavailable from four controls. Values in bold are statistically significant (*p* < 0.05). Values of *p* were derived from Pearson's Chi-squared test for categorical variables and the Student’s t test for age or the natural logarithm of other continuous variables.

ALT, alanine aminotransferase; ALP, alkaline phosphatase; anti-CTLA4, anti-cytotoxic T-lymphocyte-associated protein 4; anti-PD1, anti-programmed cell death protein 1; ChILI, checkpoint inhibitor-induced liver injury; CPI, checkpoint inhibitors; RCC, renal cell carcinoma; ULN, upper limit of normal.

∗ Mann–Whitney–Wilcoxon test was performed.

**Table 3:** Factors associated with ChILI in CPI naive patients (NUH cohort).

Factors	Unadjusted OR (95% CI)	Adjusted OR (95% CI)
Age	0.95 (0.93–0.98, *p* <0.001)	0.99 (0.95–1.02, *p* = 0.38)
Female sex	1.53 (0.76–3.05, *p* = 0.23)	2.54 (1.09–6.06, *p* = 0.03)
CPI		
Anti-PD1 (reference)	—	—
Anti-CTLA4	0.94 (0.13–4.47, *p* = 0.94)	0.65 (0.09-3.31, *p* = 0.62)
Combination followed by anti-PD1	12.88 (5.23–38.87, *p* <0.001)	10.95 (4.04-35.60, *p* <0.001)
Liver metastases	0.87 (0.29–2.15, *p* = 0.78)	1.76 (0.45–6.25, *p* = 0.39)
NLR	0.82 (0.65–0.97, *p* = 0.05)	0.90 (0.72–1.05, *p* = 0.28)
ALT	1.00 (0.99–1.01, *p* = 0.30)	1.03 (1.01–1.05, *p* = 0.006)
ALP	0.99 (0.98–1.00, *p* = 0.08)	0.99 (0.98–1.00, *p* = 0.01)

Univariable and multivariable logistic regression for ChILI occurrence. Number included in analysis = 394, number in model = 390, missing values = 4. ALT, alanine aminotransferase; ALP, alkaline phosphatase; anti-CTLA4, anti-cytotoxic T-lymphocyte-associated protein 4; anti-PD1, anti-programmed cell death protein 1; ChILI, checkpoint inhibitor-induced liver injury; CPI, checkpoint inhibitors; NLR, neutrophil to lymphocyte ratio; OR, odds ratio; ULN, upper limit of normal.

### Pattern and severity of ChILI cases from three centres

**Fig. 3:**
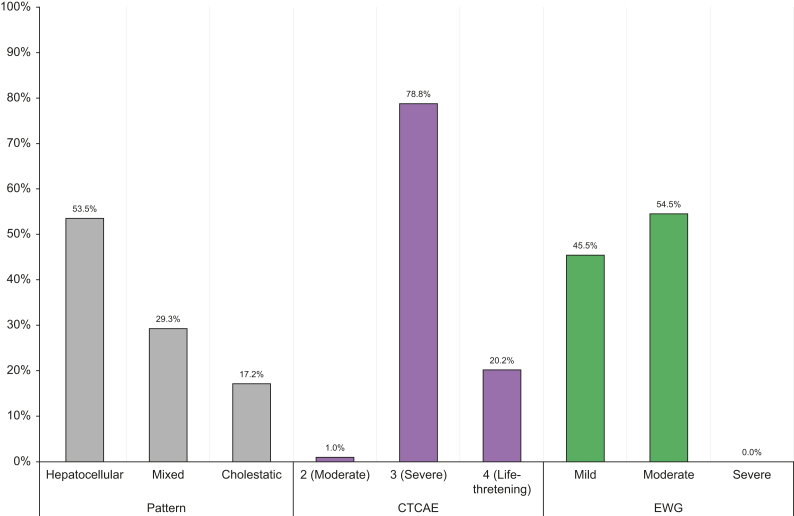
Pattern and severity of ChILI cases from three centres. The pattern of ChILI is based on the earliest identified liver chemistry elevations above the upper limit of normality (ULN) that meet ChILI criteria and defined using the R-value where R = (ALT/ULN)/(ALP/ULN). Hepatocellular (R ≥5); cholestatic (R ≤2) and mixed (R >2 and <5). ChILI, checkpoint inhibitor-induced liver injury; CTCAE, Common Terminology Criteria for Adverse Events V5.0;^8^ EWG, Expert Working Group grading in drug-induced liver injury.^12^

Consecutive patients with malignant melanoma or renal cancer with suspected ChILI at CUH and LTHT were identified and considered for inclusion. Twenty-five out of 30 from CUH and 36 out of 38 from LTHT passed adjudication. A summary of the investigations performed to exclude alternative causes and used in adjudication are shown in Table S6. Clinical characteristics of adjudicated ChILI cases from three centres are summarised in Table 4, and details of suspected cases who did not pass adjudication are summarised in Table 5. The Median RUCAM score was 7 for CUH and 8 for LTHT (probable). Among the 99 ChILI cases from three centres who passed adjudication, the pattern of liver injury was hepatocellular in 53 cases (53.5%), mixed in 29 (29.3%), and cholestatic in 17 (17.2%), as shown in Table 4 and Fig. 3. Severity grading was mild in 45 cases (45.5%) and moderate in the remaining 54 (54.5%). Out of 99, 10 patients developed jaundice and met Hy’s law (10.1%), the pattern of liver injury was hepatocellular in four, mixed in three, and cholestatic in three. However, none of the cases was severe as per EWG criteria. When compared with CTCAE criteria, 20 out of 99 ChILI cases (20.2%) were classified as grade 4 (life-threatening; urgent intervention indicated), 78 (78.8%) as grade 3 (severe or medically significant but not immediately life-threatening) and one (1%) as grade 2 (moderate) (Table 4 and Fig. 3).

**Table 4:** Clinical characteristics of ChILI cases from three centres.

	NUH (n = 38) 2011–2021	CUH (n = 25) 2014–2021	LTHT (n = 36) 2018–2021	Total (n = 99)
Cancer, n (%)				
Malignant melanoma	35 (92.1)	20 (80)	32 (88.9)	87 (87.9)
Advanced RCC	3 (7.9)	5 (20)	4 (11.1)	12 (12.1)
CPI regimen in melanoma, n (%)				
Ipilimumab + nivolumab followed by nivolumab	29 (82.9)	11 (55)	21 (65.6)	61 (70.1)
Ipilimumab	2 (5.7)	1 (5)	0	3 (3.4)
Nivolumab	0	2 (10)	2 (6.3)	4 (4.6)
Pembrolizumab	3 (8.6)	4 (20)	6 (18.8)	13 (14.9)
Adjuvant pembrolizumab	1 (2.9)	2 (10)	3 (9.4)	6 (6.9)
CPI regime in RCC, n (%)				
Ipilimumab + nivolumab followed by nivolumab	2 (66.7)	2 (40)	3 (75)	7 (58.3)
Nivolumab	1 (33.3)	3 (60)	1 (25)	5 (41.7)
Previous exposure to CPI, n (%)	2 (5.3)	3 (12)	6 (16.7)	11 (11.1)
Previous exposure chemo/targeted therapy, n (%)	6 (15.8)	7 (28)	3 (8.3)	16 (16.2)
Median number of cycles prior to ChILI (IQR)	3 (2.4)	2 (1.3)	3 (2.4)	3 (2.4)
CTCAE hepatotoxicity grade, n (%)				
2 (moderate)	1 (2.6)	0	0	1 (1)
3 (severe)	28 (73.7)	22 (88)	28 (77.8)	78 (78.8)
4 (life-threatening)	9 (23.7)	3 (12)	8 (22.2)	20 (20.2)
EWG severity grade, n (%)				
Mild	11 (28.9)	18 (72)	16 (44.4)	45 (45.5)
Moderate	27 (71.1)	7 (28)	20 (55.6)	54 (54.5)
Severe	0 (0)	0 (0)	0	0 (0)
Developed jaundice (total bilirubin ≥ twofold), n (%)	2 (5.3)	2 (8)	6 (16.7)	10 (10.1)
Pattern				
Hepatocellular	22 (57.9)	12 (48)	19 (52.8)	53 (53.5)
Mixed	11 (28.9)	8 (32)	10 (27.8)	29 (29.3)
Cholestatic	5 (13.2)	5 (20)	7 (19.4)	17 (17.2)
Median RUCAM score (IQR)	7(6.8)	7 (5.8)	8 (7.9)	8 (6.8)
Treatment of ChILI, n (%)				
Corticosteroids alone	34 (89.5)	21 (84)	19 (52.8)	74 (74.7)
Corticosteroids + MMF	2 (5.3)	2 (8)	11 (30.6)	15 (15.2)
Corticosteroids + MMF + tacrolimus	0	0 (0)	6 (16.7)	6 (6.1)
None	2 (5.3)	2 (8)	0 (0)	4 (4)
Patients with concurrent other irAE, n (%)	22 (57.9)	13 (52)	23 (63.9)	58 (58.6)
Most common concurrent irAE, n (%)				
Thyroiditis	15 (39.5)	3 (12)	2 (5.6)	20 (20.2)
Colitis	8 (21.1)	3 (12)	9 (25)	20 (20.2)
Skin rash	5 (13.2)	3 (12)	6 (16.7)	14 (14.1)
Adrenalitis	0	1 (4)	8 (22.2)	9 (9.1)
Hypophysitis	1 (2.6)	2 (8)	2 (5.6)	5 (5.1)

ChILI, checkpoint inhibitor-induced liver injury; CPI, checkpoint inhibitors; CTCAE, Common Terminology Criteria for Adverse Events V5.0;^8^ CUH, Cambridge University Hospital NHS Foundation Trust; EWG, Expert Working Group definitions and grading in drug-induced liver injury;^12^ irAE, immune-related adverse events; LTHT, Leeds Teaching Hospitals NHS Trust; MMF, mycophenolate mofetil; NUH, Nottingham University Hospitals NHS Trust; RCC, renal cell carcinoma.

**Table 5:** Characteristics of patients with suspected ChILI who were excluded after adjudication from three centres.

Age and sex	Cancer, liver metastasis prior CPI (Y/N)	CPI regime	Cycles prior liver injury	Pattern of liver injury	Received empirical steroids	Diagnosis, liver injury resolved (Y/N)	Clinical outcome at 12 months
50 F	MM (Y)	Ipilimumab + nivolumab	3	Mixed	Yes	Disease progression with widespread liver metastases (N)	Died of disease progression
51 F	MM (Y)	Ipilimumab + nivolumab	3	Hepatocellular	Yes	Disease progression with widespread liver metastases (N)	Died of disease progression
66 M	MM (Y)	Ipilimumab	2	Cholestatic	Yes	Disease progression with widespread liver metastases (N)	Died of disease progression
76 F	MM (Y)	Ipilimumab	2	Mixed	Yes	Disease progression with widespread liver metastases (N)	Died of disease progression
37 M	MM (Y)	Ipilimumab	3	Cholestatic	Yes	Liver metastases causing biliary obstruction (N)	Died of disease progression
55 F	MM (Y)	Ipilimumab	1	Hepatocellular	Yes	Disease progression with widespread liver metastases (N)	Died of disease progression
50 M	MM (Y)	Ipilimumab	4	Cholestatic	No	Disease progression with widespread liver metastases (N)	Died of disease progression
55 F	MM (Y)	Ipilimumab	2	Cholestatic	Yes	Disease progression with widespread liver metastases (N)	Died of disease progression
59 M	MM (Y)	Ipilimumab	4	Cholestatic	No	Disease progression with widespread liver metastases (N)	Died of disease progression
73 F	RCC (N)	Nivolumab	14	Cholestatic	No	DILI (Y)	Nivolumab was restarted with no elevation in liver enzymes
75 M	MM (N)	Adjuvant pembrolizumab	1	Cholestatic	Yes	Disease progression with widespread liver metastases (N)	Died of disease progression
63 M	MM (N)	Adjuvant pembrolizumab	1	Mixed	Yes	Disease progression with widespread liver metastases (N)	Died of disease progression
75 F	MM (N)	Pembrolizumab	9	Cholestatic	Yes	Cholangitis/biliary obstruction (Y)	Pembrolizumab was restarted with no elevation in liver enzymes
69 M	MM (Y)	Ipilimumab + nivolumab	1	Cholestatic	Yes	Disease progression with widespread liver metastases (N)	Died of disease progression
62 M	MM (Y)	Ipilimumab + nivolumab	1	Hepatocellular	Yes	Disease progression with widespread liver metastases (N)	Died of disease progression
76 F	MM (Y)	Ipilimumab + nivolumab	1	Mixed	Yes	Disease progression with widespread liver metastases (N)	Died of disease progression

ChILI, checkpoint inhibitor-induced liver injury; CPI, checkpoint inhibitors; DILI, idiosyncratic drug-induced liver injury; F, female; M, male; MM, malignant melanoma; RCC, renal cell carcinoma.

### Treatment of ChILI, resolution, and association with other irAE

Out of 99 patients who developed ChILI from three centres, only four patients did not receive corticosteroids (4%). Furthermore, out of 16 patients who were suspected to have ChILI but had alternative aetiology after investigations, 13 were empirically treated with corticosteroids (81%). Fifty-one patients out of 99 were hospitalised following oncology guidelines (51.5%). All hospitalised patients received corticosteroids, most received i.v. methylprednisolone, 38 out of 51 patients (74.5%). The rest of the hospitalised patients received oral prednisolone; the dose varied from 50 to 225 mg once daily. Twenty-one out of 99 patients received second-line immunosuppression (21.3%), 15 had mycophenolate mofetil (MMF) and six patients received MMF plus tacrolimus. There was obvious variability in management strategies between centres, as highlighted in Table 4. Nine out of 99 patients underwent liver biopsy as part of investigations and the histological features were consistent with ChILI. Five out of 36 ChILI cases who received steroids at NUH (13.9%), had clinically significant side effects related to corticosteroid therapy; two developed diabetes, two developed myopathy, and one had a severe acneiform rash. Steroid-related adverse events were not recorded at other centres.

The median time to resolution of ChILI in patients treated with steroids was 67 days (range 11–550 days) and time to first liver enzymes ≤ grade 1 CTCAE was 21 days (range 2–133 days). In the four patients untreated with steroids, median time to resolution was 34 days (range 28–63 days) and time to first liver enzymes ≤ grade 1 CTCAE was 24 days (range 15–28 days). The difference in time to resolution and to ≤ grade 1 CTCAE toxicity was not statistically significant between CPI classes (Figs S1–S4).

Among patients with ChILI, 58 developed other concurrent irAE (58.6%). Thyroiditis and colitis were the most common, occurring in 20 patients (20.2%) followed by skin rash in 14 (14.1%), adrenalitis in nine (9.1%) and hypophysitis in five (5.1%) (Table 4).

### Outcome of rechallenge

Rechallenge was performed in 37 out of 99 patients (37.4%), 31 of those had ChILI following combination therapy, four following pembrolizumab, and one each after nivolumab monotherapy and ipilimumab monotherapy. Thirty-five patients received anti-PD1 monotherapy as a rechallenge CPI regime, one had ipilimumab monotherapy, and one had combination therapy. Details of CPI regimes used in rechallenge and recurrence are summarised in Table S7. Three patients were taking immunosuppression at the time of rechallenge (oral prednisolone 10–25 mg), one as a prophylactic measure for ChILI, one was on a steroids-tapering regime, and one received it for previous CPI-induced pneumonitis.

Seven patients developed elevation of liver enzymes on rechallenge (21.6%). Three patients met EWG criteria (8.1%) which was defined as recurrence of ChILI. Four patients developed grade 2 hepatotoxicity that did not meet DILI criteria and CPI was permanently stopped (ALT elevation three-to five-fold without rise in bilirubin). Nine patients developed other clinically significant irAE requiring hospitalisation or treatment (24.3%); five had colitis, two had hypophysitis, one had severe neuropathy, and one had skin toxicity.

## Discussion

We have established the risk, incidence rate, and cumulative probability of ChILI in patients with malignant melanoma or advanced RCC, per cancer and CPI regime in a real-world cohort over 10 years. All ChILI cases were defined and graded using DILI EWG recommendation and formally adjudicated following causality assessment.^12^^,^^23^ The overall risk of ChILI was 8.8% and the incidence rate was 11.5 per 1,000 person-months. The highest risk and incidence occurred in patients with melanoma receiving a combination of anti-CTLA4 and anti-PD1, 28.4% and 38.1 per 1,000 person-months, respectively. Therefore, the risk of ChILI in clinical practice even when stringent criteria applied is higher than previously reported in clinical trials.^9^^,^^14^ Patients with melanoma are the most common group to be affected by ChILI and this is explained by the fact that they frequently receive combination CPI therapy. Combination therapy has been associated with higher frequency of hepatotoxicity in clinical trials and the Dutch national registry.^2^^,^^9^^,^^17^^,^^33^ The overall risk of ChILI of 8.8% among those treated with CPI is remarkably high when compared with an estimated incidence of amoxicillin–-clavulanate DILI (a common aetiology) of 0.16% among those exposed to the drug.^34^ The magnitude of difference in the incidence of DILI and ChILI may well be related to the underlying mechanism. Genetic and experimental medicine studies that have investigated DILI support the ‘hapten mechanism’ where MHC class II and I restricted CD4+ and CD8+ clones are activated by a drug-derived antigen.^35^ We have recently identified a distinct population of circulating effector memory CD8^+^ cells in patients during an acute phase of ChILI.^36^ CPI act upon T cell surface receptors as their therapeutic target which may explain in part the high incidence of irAE such as ChILI. Although rs16906115 near the *IL7* locus has been associated with irAE in a genome-wide association study,^37^ the variant has not been associated with liver-related adverse events in particular. Robust case definitions and causality assessments are essential to identify risk factors for ChILI.

The cumulative probability of ChILI in patients who received combination therapy followed by anti-PD1 was 32% with a median latency of 51 days from the start of CPI. ChILI occurred as early as 17 days after the first cycle of ipilimumab and nivolumab, with no new events noted beyond 135 days for patients who continued nivolumab monotherapy. These data indicate that in this group of patients, the risk of ChILI falls in the later phase of treatment and therefore intensity of monitoring may be reduced accordingly. In contrast, in patients who received anti-PD1 monotherapy (nivolumab or pembrolizumab), ChILI developed after a median of 106 days, with one patient developing ChILI after 1 year of exposure. Interestingly, the cumulative probability of ChILI following anti-CTLA4 was similar to anti-PD1 (2.9% compared with 2.5%) contrary to previous suggestions.^13^

We assessed risk factors associated with ChILI occurrence in CPI-naive patients. CPI regime, female sex, and baseline ALP and ALT were significant independent risk factors associated with ChILI. Patients starting combination therapy with anti-CTLA4 and anti-PD1 have a significantly increased risk of ChILI compared with patients receiving anti-PD1 monotherapy, this is consistent with previous studies.^4^ Furthermore, women were almost three times more likely to develop ChILI than men. In recent study that involved 877 patients of patients undergoing CPI, women had 54% increased risk of symptomatic adverse reaction.^20^ In another study of 1,096 patients treated with CPI, liver toxicity was significantly more common in women;^21^ however, multivariable modelling was not performed to confirm independent association. Sex differences in efficacy, toxicity, and increased susceptibility of women to toxicity from chemotherapy in particular have been observed before.^38^ However, whether these are attributable to differences in tumour biology or pharmacokinetics/pharmacodynamics of CPI are yet to be elucidated. Further understanding of the impact of sex on CPI therapy and irAE is needed.^20^^,^^38^

An increase of baseline ALT was a risk factor for the development of ChILI; a similar observation was made in a recent study of anti-tuberculosis DILI.^39^ Intriguingly, higher baseline ALP was associated with reduced ChILI occurrence. Although these two associations might not be clinically significant, they may reflect important mechanistic associations. In a mice model, ALP protected the animal from acute liver injury induced by delayed-type hypersensitivity.^40^ It has also been shown that ALP is able to detoxify lipopolysaccharide derived from the gut–portal axis by removing the terminal phosphate group, thus limiting lipopolysaccharide-mediated liver injury.^41^^,^^42^ Further research is needed to explore the potential mechanisms by which intestinal microbiota influences susceptibility to ChILI.^43^

Age and presence of liver metastases at baseline were not independent risk factor of ChILI, which is consistent with previous studies.^17^^,^^44^ A retrospective cohort showed that the incidence of all grade liver toxicity is highest in patients 50–64 years old (10.6%) compared with 3.1% in the 65–74 age group and 4.3% in the age group >74; however, the difference did not reach statistical significance.^44^ Another retrospective study reported a comparable proportion of liver metastases between patients with and without ChILI.^17^ Although baseline NLR was significantly different between patients with and without ChILI, a similar observation recently reported,^21^ it was not independently associated with ChILI in multivariable analysis. This highlights the importance of using appropriate statistical models to evaluate independent associations.

We identified all patients who developed acute liver injury among those treated at NUH over a 10-year period. Of those who met the predetermined criteria, 89.4% (42 out of 47) started promptly on steroids for suspected ChILI. Nonetheless, six of those treated with steroids (14.3%) were found to have an alternative explanation for liver biochemistry abnormalities. Furthermore, all patients with suspected ChILI from the other two centres who did not pass adjudication because of alternative aetiology after work-up received empirical corticosteroids. It can be argued that both the interruption of CPI and introduction of steroids were not necessary in these patients. A similar yield of alternative diagnoses has been reported in other studies.^11^^,^^45^^,^^46^ These observations stress the importance of careful evaluation of suspected ChILI and causality assessment before initiating steroid therapy as much as possible. Only 8% of ChILI cases with available antinuclear antibodies had a positive result and only one patient had elevated level of serum immunoglobulin G (IgG). Riveiro-Barciala *et al.*^46^ reported higher proportion of available antinuclear antibodies positivity (32%) among ChILI cases but also reported one patient with raised IgG highlighting the difference in prevalence of autoantibodies in ChILI compared with autoimmune hepatitis.

In addition, a fifth of ChILI patients from three centres were classified as grade 4 CTCAE hepatotoxicity (life-threatening), whereas only 10% of all cases developed jaundice and none developed acute liver failure. Over half of patients were hospitalised as CTCAE recommends hospitalisation in patients with hepatitis grade 3 and higher.^8^ Rapid evaluation of patients with suspected ChILI ahead of initiation of steroids should be considered. It is desirable that currently used definitions and grading of liver injury after CPI are revised in view of these findings.

European Society of Medical Oncology (ESMO) and the American Society of Clinical Oncology (ASCO) guidelines recommend steroid therapy for ≥ grade 2 hepatitis.^47^^,^^48^ The vast majority (96%) of patients who developed ChILI from three cohorts received corticosteroids, similar to other studies.^46^ This reflects the current practice despite the growing evidence that some patients improve without the need for immunosuppression.^49^^,^^50^ Corticosteroid therapy was associated with significant side effects that required medical attention in 13% of ChILI cases in our cohort. Steroid therapy in the management irAE has been shown to increase the risk of serious infections and osteopaenia.^51^^,^^52^ The number of ChILI cases who did not receive steroids was very small so no valid conclusions can be drawn regarding steroid use or duration to resolution of liver injury. Another study that compared patients with hepatotoxicity following CPI (based on CTCAE criteria) who received steroids (n = 67) with those not receiving steroids (n = 33) showed that the median duration of improvement of ALT was significantly shorter in patients without steroids;^50^ there were no differences in any of the clinical characteristics observed between these two groups other than steroid therapy. In addition, there are multiple reports of liver injury attributed to corticosteroids themselves including oral prednisolone,[53], [54], [55] and methylprednisolone.^56^ Furthermore, the use of second- and third-line immunosuppression varied significantly between centres despite no cases with acute liver failure. Therefore, future prospective studies involving a large number of well-phenotyped cases of ChILI are needed to inform the optimum management of ChILI including risks and benefits of corticosteroids and other immunosuppressive drugs.

More than a third of patients with ChILI were rechallenged with anti-PD1 monotherapy being the most common CPI class used. A total of 21.6% developed elevation of liver enzymes following rechallenge and CPI was stopped, consistent with other studies.^13^^,^^50^ However, only 8.1% met EWG criteria at the time of recurrence of liver injury. Several prophylactic strategies have been proposed when considering rechallenging following irAE affecting other organs;^57^ however, there is limited evidence specific to hepatotoxicity. Further research is needed to investigate mechanisms, risk factors, and possible effective prophylactic strategies to predict and avoid recurrence of ChILI.

Our study differs from previously published retrospective studies. Our cohort is homogenous and included two main cancer populations who receive CPI as standard of care without concomitant oncological treatments. We included all patients who received CPI over 10 years to enable accurate estimation of the frequency of ChILI and minimise selection bias. In another retrospective study that reported 28 patients from 15 different cancers who developed ChILI, nine of them (32%) received concomitant oncological treatment in addition to CPI.^46^ To our knowledge, this is the only study that defined ChILI based on DILI criteria, systematically used a prescription event monitoring method in a 10-year cohort and investigated independent associations in multivariable analysis of risk factors.

Despite its strengths, our study has some limitations. To calculate the incidence rate, the time at risk after stopping CPI, for patients who did not develop ChILI, was estimated based on CPI regime frequency and published data on the clinical pharmacokinetics and pharmacodynamics of CPI.^26^ However, this might underestimate the duration of T cell activation after stopping CPI which lasts beyond the half-life of the CPI and varies between patients. Patients were followed up for a minimum of 90 days after stopping CPI. In a literature review that included 194 trials and 367 case reports and series from 2008 to 2018, the incidence of delayed ChILI presenting ≥90 days after discontinuation of immunotherapy was limited to one case over a period of 10 years.^58^ This is consistent with our findings too. All patients who were receiving CPI had a minimum of 6 months’ exposure of CPI before the data collection cut-off. We acknowledge that our risk factor model had small sample size and further validation in large independent cohort is desirable.

In conclusion, the overall incidence rate of ChILI in malignant melanoma and advanced RCC patients is 11.5 per 1,000 person-months. Melanoma patients receiving combination therapy have the highest risk of ChILI; however, the risk of new-onset ChILI in melanoma and renal cancer patients who received combination therapy and continued on anti-PD1 drops after 4.5 months of therapy. Women and patients with low baseline ALP and high baseline ALT have an increased risk of developing ChILI. Delineation of the period at which adverse events appear during the course of CPI therapy, use of standardised criteria to define ChILI and structured evaluation using validated causality assessment would harmonise care of these patients.

## Financial support

This work was supported by the National Institute of Health Research Nottingham Biomedical Research Centre (BRC-1215-20003) and the 10.13039/501100010767Innovative Medicines Initiative 2 Joint Undertaking under grant agreement No 821283 (www.imi.europa.eu). This Joint Undertaking receives the support from the 10.13039/501100007601European Union’s Horizon 2020 research and innovation programme and EFPIA. The funders had no role in study design, data collection and analysis, decision to publish, or preparation of the manuscript.

## Authors’ contributions

Manuscript preparation and writing: EA. Manuscript revision: GPA. Statistical analyses: EA, CC. Contributed to data collection: AO, ID, AH, JK, CL, ASK. Contributed to the text of the manuscript: SJW, BO, CRC, AR, HF, PMP. All authors have reviewed and approved the final submitted manuscript.

## Data availability statement

The data that support the findings of this study are available on reasonable request to the corresponding author.

## Conflicts of interest

GPA has received consulting fees from Pfizer, GlaxoSmithKline, Clinicpace, Servier Pharmaceuticals, NuCANA Plc, AstraZeneca, and BenevolentAI paid to the University of Nottingham. All authors declare no conflicts of interest that relate to this work.

Please refer to the accompanying ICMJE disclosure forms for further details.
