# Supplementary material for: Incidence, risk factors and outcomes of checkpoint inhibitor-induced liver injury: A 10-year real-world retrospective cohort study
Source: JHEP Rep. 2023 Jul 18;5(10):100851. doi: 10.1016/j.jhepr.2023.100851 (PMC10505983; doi:10.1016/j.jhepr.2023.100851)
Supplement: Multimedia component 3 [file mmc3.pdf]

Incidence, risk factors and outcomes of checkpoint inhibitor-induced liver injury: A 10-year real-world retrospective cohort study

Edmond Atallah, Sarah J. Welsh, Brent O'Carrigan, Ana Oshaughnessy, Igboin Dolapo, Andrew S. Kerr, Joanna Kucharczak, Colin Lee, Colin Crooks, Amy Hicks, Chenchu Ramu Chimakurthi, Ankit Rao, Hester Franks, Poulam M. Patel, Guruprasad P. Aithal

Table of contents

Supplementary figures....2

Supplementary tables....4

Supplementary references....9

Figures

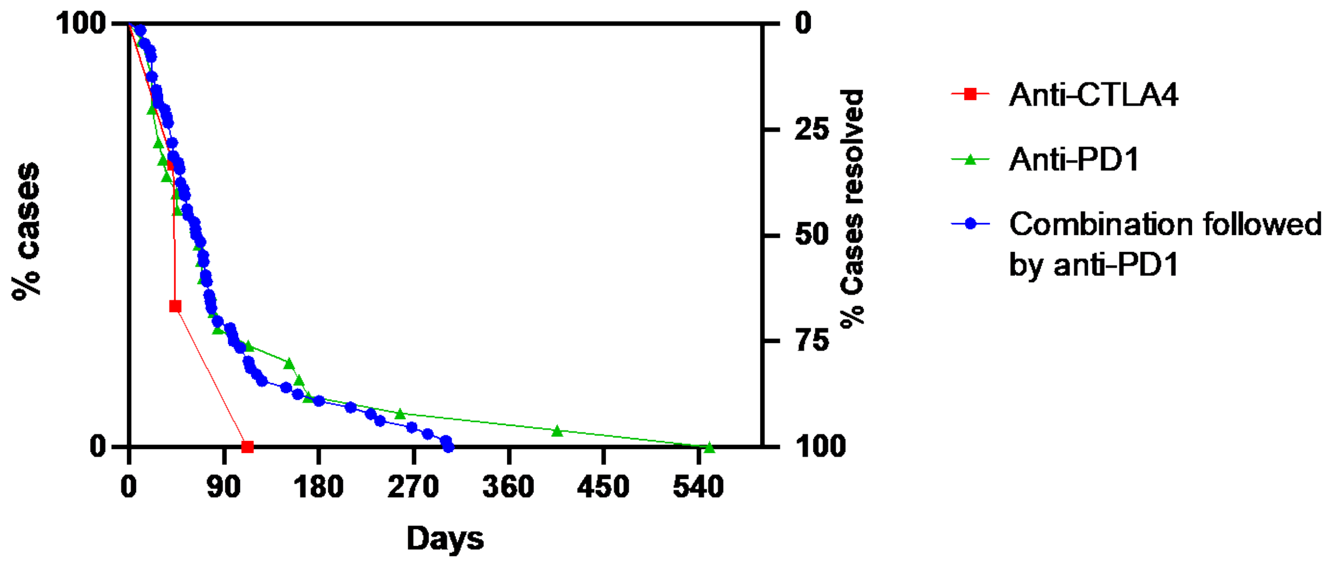

Fig S1. Time to resolution of checkpoint inhibitor-induced liver injury (back to baseline or normalisation) in each CPI class. Cases that fully resolved are plotted (n=92); anti-CTLA4 (n=3), anti-PD1 (n=25), combination followed by anti-PD1 (n=64). Abbreviations: Anti-CTLA4, Anti-cytotoxic T-lymphocyte-associated protein 4; Anti-PD1, Anti-programmed cell death protein 1.

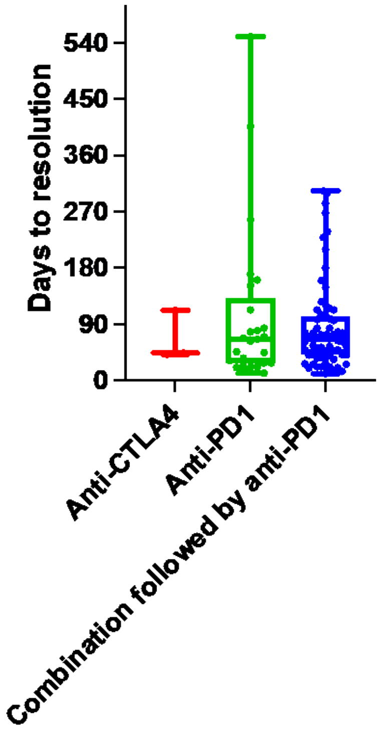

Fig S2. Box and whiskers graph illustrating median time and range to resolution of checkpoint inhibitor-induced liver injury (back to baseline or normalisation) in each CPI class. Cases that fully resolved are plotted (n=92); anti-CTLA4 (n=3), anti-PD1 (n=25), combination followed by anti-PD1 (n=64). Kruskal-Wallis statistic: 0.097, p=0.95. Abbreviations: Anti-CTLA4, Anti-cytotoxic T-lymphocyte-associated protein 4; Anti-PD1, Anti-programmed cell death protein 1.

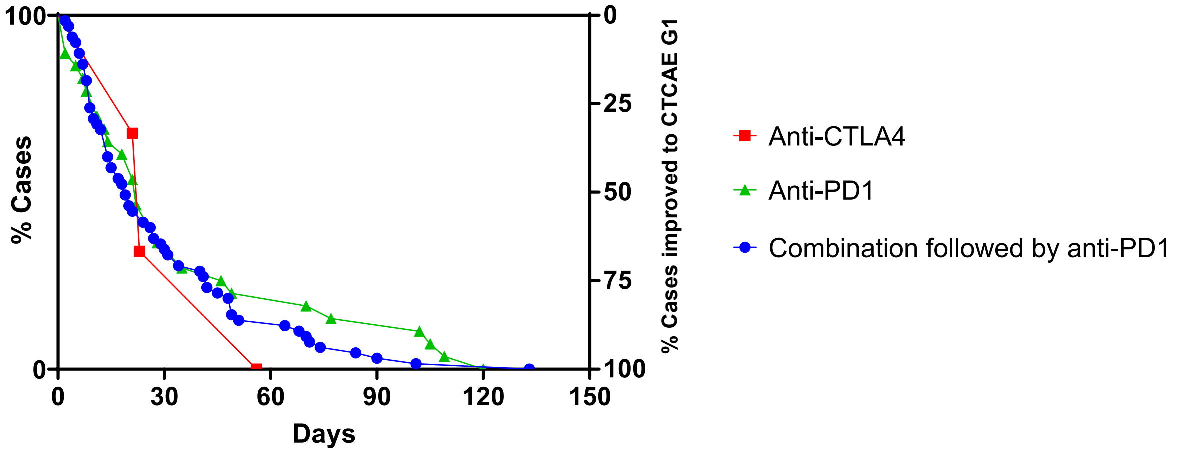

Fig S3. Time to improvement to ≤ CTCAE grade 1 in each CPI class. Number of cases plotted (n=96); anti-CTLA4 (n=3), anti-PD1 (n=28), combination followed by anti-PD1 (n=65). Abbreviations: Anti-CTLA4, Anti-cytotoxic T-lymphocyte-associated protein 4; Anti-PD1, Anti-programmed cell death protein 1.

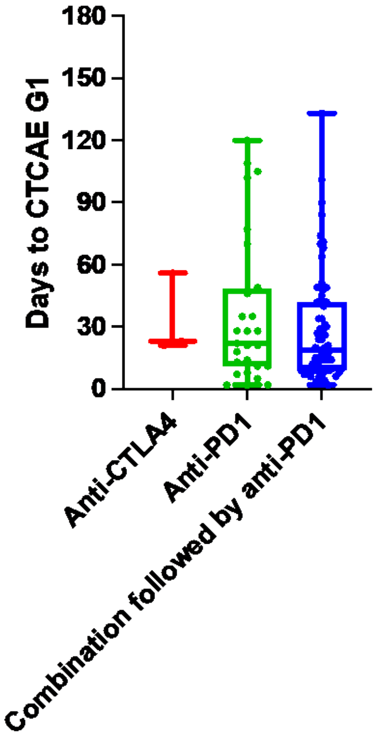

Fig S4. Box and whiskers graph illustrating median time and range to first improvement to CTCAE grade 1 in each CPI class. Number of cases plotted (n=96); anti-CTLA4 (n=3), anti-PD1 (n=28), combination followed by anti-PD1 (n=65). Kruskal-Wallis statistic: 0.9364, p=0.6. Abbreviations: Anti-CTLA4, Anti-cytotoxic T-lymphocyte-associated protein 4; Anti-PD1, Anti-programmed cell death protein 1.

Tables

Table S1. Definitions of hepatotoxicity according to the National Cancer Institute criteria compared to expert working group criteria in drug-induced liver injury

**Liver enzymes**	**CTCAE**				**EWG**
	Grade 1 Mild	Grade 2 Moderate	Grade 3 Severe	Grade 4 Life-threatening	
**ALT (xULN)**	>1.0-3.0	>3.0-5.0	>5.0-20	>20	≥5 OR ≥3 AND > 2xULN TB
**ALP (xULN)**	>1.0-2.5	>2.5-5.0	>5.0-20	>20	≥2
**TB (xULN)**	>1.0-1.5	>1.5-3.0	>3.0-10	>10	-

Abbreviations: ALT, Alanine aminotransferase; ALP, Alkaline phosphatase; TB, Total bilirubin; ULN, Upper limit of normal; CTCAE, Common Terminology Criteria for Adverse Events V5.0 (1); EWG, Expert Working Group definitions and grading in drug-induced liver injury (2)

Table S2. Frequency and dose of CPI regimes

<table>
<tr><th>Cancer</th><th>CPI Regime</th><th colspan="2">Frequency and dose</th></tr>
<tr><td rowspan="5">Malignant Melanoma</td><td>Ipilimumab</td><td colspan="2">3 mg/kg every 3 weeks for up to 4 cycles</td></tr>
<tr><td>Ipilimumab + Nivolumab followed by Nivolumab</td><td><u>Combination phase:</u>
Ipilimumab: 3 mg/kg + nivolumab 1 mg/kg every 3 weeks for up to 4 cycles</td><td><u>Monotherapy phase:</u>
Nivolumab 240 mg every 2 weeks or 480 mg every 4 weeks</td></tr>
<tr><td>Nivolumab</td><td colspan="2">240 mg every 2 weeks or 480 mg every 4 weeks</td></tr>
<tr><td>Pembrolizumab</td><td colspan="2">400 mg every 6 weeks or 200 mg every 3 weeks</td></tr>
<tr><td>Adjuvant Pembrolizumab</td><td colspan="2">400 mg every 6 weeks or 200 mg every 3 weeks</td></tr>
<tr><td rowspan="2">Advanced RCC</td><td>Ipilimumab + Nivolumab followed by Nivolumab</td><td><u>Combination phase:</u>
Ipilimumab: 1 mg/kg + nivolumab 3 mg/kg every 3 weeks for up to 4 cycles</td><td><u>Monotherapy phase:</u>
Nivolumab 240 mg every 2 weeks or 480 mg every 4 weeks</td></tr>
<tr><td>Nivolumab</td><td colspan="2">240 mg every 2 weeks or 480 mg every 4 weeks</td></tr>
</table>

Abbreviations: CPI, Checkpoint inhibitors; RCC, Renal cell carcinoma.

Table S3. Cumulative probability of ChILI in melanoma and renal cancer patients receiving combination therapy followed by anti-PD1 over 1 year

Time point (days)	Probability	Variance	Lower CI	Upper CI	Number at risk	Number of events
**0**	-	-	-	-	121	-
**17**	0.8%	6.77E-05	0.1%	5.7%	121	1
**19**	2.5%	2.00E-04	0.8%	7.5%	120	2
**21**	3.3%	2.64E-04	1.3%	8.6%	118	1
**24**	5.0%	3.90E-04	2.3%	10.7%	117	2
**28**	5.8%	4.51E-04	2.8%	11.8%	115	1
**32**	6.8%	5.38E-04	3.4%	13.1%	95	1
**33**	7.8%	6.24E-04	4.1%	14.4%	94	1
**34**	8.8%	7.08E-04	4.8%	15.7%	93	1
**35**	9.8%	7.90E-04	5.5%	17.0%	92	1
**39**	10.7%	8.70E-04	6.2%	18.2%	91	1
**42**	11.7%	9.48E-04	7.0%	19.4%	90	1
**47**	12.7%	1.02E-03	7.7%	20.6%	89	1
**49**	14.7%	1.17E-03	9.3%	23.0%	88	2
**50**	15.8%	1.25E-03	10.1%	24.2%	81	1
**51**	16.8%	1.33E-03	10.9%	25.4%	80	1
**52**	17.9%	1.41E-03	11.8%	26.7%	78	1
**56**	18.9%	1.48E-03	12.6%	27.9%	77	1
**59**	20.0%	1.56E-03	13.5%	29.2%	75	1
**61**	21.1%	1.63E-03	14.4%	30.4%	74	1
**65**	22.2%	1.70E-03	15.3%	31.6%	73	1
**69**	23.3%	1.77E-03	16.2%	32.8%	72	1
**77**	24.4%	1.85E-03	17.1%	34.1%	68	1
**87**	25.5%	1.92E-03	18.1%	35.3%	66	1
**92**	26.7%	2.00E-03	19.1%	36.7%	63	1
**105**	28.0%	2.09E-03	20.1%	38.1%	58	1
**111**	29.3%	2.18E-03	21.2%	39.6%	55	1
**126**	30.7%	2.28E-03	22.4%	41.1%	52	1
**135**	32.1%	2.39E-03	23.6%	42.7%	48	1

Abbreviations: Anti-PD1, Anti-programmed cell death protein 1; ChILI, Checkpoint inhibitor-induced liver injury; CI, Confidence interval.

Table S4. Cumulative probability of ChILI in in melanoma patients receiving anti-CTLA4 monotherapy over 1 year

Time point (days)	Probability	Variance	Lower CI	Upper CI	Number at risk	Number of events
**0**	-	-	-	-	88	-
**26**	1.4%	0.0002	0.2%	9.2%	74	1
**46**	2.9%	0.0004	0.7%	11.2%	63	1

Abbreviations: Anti-CTLA4, Anti-cytotoxic T-lymphocyte-associated protein 4; ChILI, Checkpoint inhibitor-induced liver injury; CI, Confidence interval.

Table S5. Cumulative probability of ChILI in in melanoma and renal cancer patients receiving anti-PD1 monotherapy over 1 year

Time point (days)	Probability	Variance	Lower CI	Upper CI	Number at risk	Number of events
**0**	-	-	-	-	223	-
**70**	0.5%	3.00E-05	0.1%	3.8%	182	1
**82**	1.1%	5.97E-05	0.3%	4.3%	181	1
**106**	1.7%	9.74E-05	0.6%	5.3%	159	1
**166**	2.5%	1.55E-04	0.9%	6.6%	127	1

Abbreviations: Anti-PD1, Anti-programmed cell death protein 1; ChILI, Checkpoint inhibitor-induced liver injury; CI, Confidence interval.

Table S6. List of investigations performed to exclude alternative causes in the 99 ChILI cases and were available for adjudication

Liver investigation		Proportion of ChILI cases
**Imaging**		100%
**Modality of liver imaging used**	Ultrasound liver	57%
	CT scan	41%
	MRI Liver	2%
**Auto-immune liver screen**	Anti-Nuclear Antibodies (ANA)	67%
	Anti-Smooth Muscle Antibodies (ASMA)	56%
	Anti-Mitochondrial Antibodies (AMA)	59%
	Liver-Kidney Microsomal Antibodies (LKM)	58%
	Immunoglobulin G (IGG)	59%
	Immunoglobulin A (IGA)	59%
	Immunoglobulin M (IGM)	59%
**Virology screen**	Hepatitis A (IGM anti-HAV)	73%
	Hepatitis B (HBsAg and/or HBV DNA)	86%
	Hepatitis C (Anti-HCV and/or HCV DNA)	86%
	Hepatitis E (IGM anti-HEV and/or HEV RNA)	74%
	CMV (IGM anti-CMV and/or CMV PCR)	70%
	EBV (IGM anti-EBV and/or EBV DNA)	69%
**Liver Biopsy**		9%

Abbreviations: ChILI, Checkpoint inhibitor-induced liver injury; HAV, Hepatitis A virus; HBV, Hepatitis B virus; HCV, Hepatitis C virus; CMV, Cytomegalovirus; EBV, Epstein–Barr virus

Table S7. Characteristics of ChILI patients who were rechallenged

<table>
<tr><th colspan="2" rowspan="2"></th><th rowspan="2">ChILI patients, n</th><th colspan="2">ChILI patients rechallenged (n=37)</th><th rowspan="2">Recurrence of ChILI (n=3)</th></tr>
<tr><th>n(%)</th><th>Rechallenge regime used</th></tr>
<tr><td rowspan="2">Cancer</td><td>Malignant Melanoma</td><td>87</td><td>33 (37.9%)</td><td></td><td>2</td></tr>
<tr><td>Advanced RCC</td><td>12</td><td>4 (33.3%)</td><td></td><td>1</td></tr>
<tr><td rowspan="8">Initial CPI regime in Melanoma</td><td rowspan="2">Ipilimumab + Nivolumab followed by Nivolumab</td><td rowspan="2">61</td><td rowspan="2">28 (45.9%)</td><td>Nivolumab (n=27)</td><td>1</td></tr>
<tr><td>Ipilimumab + Nivolumab (n=1)</td><td>0</td></tr>
<tr><td>Ipilimumab monotherapy</td><td>3</td><td>1 (33.3%)</td><td>Pembrolizumab</td><td>0</td></tr>
<tr><td>Nivolumab monotherapy</td><td>4</td><td>0 (0.0%)</td><td></td><td>0</td></tr>
<tr><td rowspan="2">Pembrolizumab</td><td rowspan="2">15</td><td rowspan="2">4 (26.7%)</td><td>Pembrolizumab (n=3)</td><td>1</td></tr>
<tr><td>Ipilimumab (n=1)</td><td>0</td></tr>
<tr><td>Adjuvant Pembrolizumab</td><td>4</td><td>0 (0.0%)</td><td></td><td>0</td></tr>
<tr><td colspan="5"></td></tr>
<tr><td rowspan="2">Initial CPI regime in RCC</td><td>Ipilimumab + Nivolumab followed by Nivolumab</td><td>7</td><td>3 (42.9%)</td><td>Nivolumab</td><td>1</td></tr>
<tr><td>Nivolumab monotherapy</td><td>5</td><td>1 (20.0%)</td><td>Nivolumab</td><td>0</td></tr>
<tr><td rowspan="3">CTCAE grade of hepatotoxicity</td><td>2</td><td>1</td><td>1 (100.0%)</td><td></td><td>Developed recurrence (grade 3 CTCAE)</td></tr>
<tr><td>3</td><td>78</td><td>30 (38.5%)</td><td></td><td>1 developed recurrence (grade 4 CTCAE)</td></tr>
<tr><td>4</td><td>20</td><td>6 (30.0%)</td><td></td><td>1 developed recurrence (grade 3 CTCAE)</td></tr>
</table>

Abbreviations: CPI, Checkpoint inhibitors; CUH, Cambridge University Hospitals; ChILI, Checkpoint inhibitor-induced liver injury; CPI, Checkpoint inhibitors; CTCAE, Common Terminology Criteria for Adverse Events V5.0 (1) RCC, Renal cell carcinoma.

Supplementary References

1. National Cancer Institute. Common Terminology Criteria for Adverse Events (CTCAE) Version 5.0: National Cancer Institute; 2017 [78]. Available from: https://ctep.cancer.gov/protocoldevelopment/electronic_applications/docs/CTCAE_v5_Quick_Reference_8.5x11.pdf.
2. Aithal GP, Watkins PB, Andrade RJ, Larrey D, Molokhia M, Takikawa H, et al. Case definition and phenotype standardization in drug-induced liver injury. Clin Pharmacol Ther. 2011;89(6):806-15.
